# Metronidazole- and Amoxicillin-Loaded PLGA and PCL Nanofibers as Potential Drug Delivery Systems for the Treatment of Periodontitis: In Vitro and In Vivo Evaluations

**DOI:** 10.3390/biomedicines9080975

**Published:** 2021-08-07

**Authors:** Shahla Mirzaeei, Mahla Mansurian, Kofi Asare-Addo, Ali Nokhodchi

**Affiliations:** 1 Pharmaceutical Sciences Research Center, Health Institute, Kermanshah University of Medical Sciences, Kermanshah 6715847141, Iran; 2 Nano Drug Delivery Research Center, Health Technology Institute, Kermanshah University of Medical Sciences, Kermanshah 6715847141, Iran; 3 Student Research Committee, Kermanshah University of Medical Sciences, Kermanshah 6715847141, Iran; mahla.mansurian@yahoo.com; 4 Department of Pharmacy, University of Huddersfield, Huddersfield HD1 3DH, UK; k.asare-addo@hud.ac.uk; 5 Pharmaceutics Research Laboratory, School of Life Sciences, University of Sussex, Brighton BN1 9RH, UK

**Keywords:** periodontitis, metronidazole, amoxicillin, nanofiber, intrapocket, drug release

## Abstract

The purpose of this study was to prepare poly (D-L) lactide-co-glycolide (PLGA) and poly ε-caprolactone (PCL) nanofibers containing metronidazole and amoxicillin using an electrospinning process as intrapocket sustained-release drug delivery systems for the treatment of periodontal diseases. Scanning electron microscopy showed that the drug containing PLGA and PCL nanofibers produced from the electrospinning process was uniform and bead-free in morphology. The obtained nanofibers had a strong structure and resisted external tension according to the tensiometry results. The cytotoxicity results indicated acceptable cell viability (>80%). Quantification by high-performance liquid chromatography showed almost complete in vitro drug release between 7 and 9 days, whereas 14 days were required for complete drug release in vivo. No significant signs of irritation or inflammatory reaction were detected after three weeks of subcutaneous implantation of nanofibers in the animal models, thus indicating suitable compatibility. The results therefore suggest that the designed nanofibers can be used as potential commercial formulations in the treatment of periodontitis as controlled-release intrapocket drug delivery systems that can increase patient compliance. This is due to their ability to reduce the frequency of administration from three times daily in a systemic manner to once weekly as local delivery.

## 1. Introduction

Periodontitis refers to any inflammatory and infectious condition affecting one or more parts of the periodontium. The main pathogenesis of periodontal diseases is subgingival plaque formation, which can be caused by up to 400 species of bacteria. *Aggregatibacter actinomycetemcomitans*, *Porphyromonas gingivalis*, and *Fusobacter nucleatum* are common species that can invade the tissue of the periodontium [1]. There are also some inflammatory biomarkers that play important roles in the progression of periodontal diseases, such as TNF-α and different cytokines [2,3]. Treatment of periodontitis is dependent on possible pathogenesis mechanisms and preventive lifestyle modifications, such as good oral hygiene and scheduled examinations [4,5]. Generally, there are various therapies to treat periodontal disease, such as surgical and nonsurgical, resective and remodeling, and empirical, but the selection of the proper treatment is based on the decision of a specialist [6].

Nonsurgical protocols are usually recommended for first-line therapy in mild to moderate conditions, in which scaling and root planning (SRP) is a common method of treatment. In this method, all of the possible factors involved in inflammation and infection are removed from the surface of the root and periodontal space around a tooth [7].

Antibiotic therapy is necessary for the eradication of microbial plaque because of its important role in the progression of periodontitis [8]. One of the common methods is the use of combination therapy (combination of amoxicillin with metronidazole). This is especially effective against aggressive types of periodontitis [9,10]. Combination therapy of metronidazole with amoxicillin in SRP showed excellent clinical outcomes thanks to the significant reduction in the treatment time and positive effects on diagnostic factors including probing depth (PD), gingival index (GI), bleeding on probing (BOP), and plaque index (PI) [11].

Surgical therapies are the most effective method available to treat severe types of periodontal diseases. In this method, dentists focus on reducing gingival depth or removing residual parts that can cause recurrences. Surgery can also be used to repair any possible losses due to long-term inflammation [12].

Local drug delivery systems reduce systemic adverse effects as well as providing appropriate therapeutic concentrations. They have no first-pass effect or rapid onset and can also prevent microbial resistance against certain bacterial species [13]. Their limitations, however, include allergic reactions, lower efficacy in comparison to surgical methods, and challenges in implantation [14].

The ability to act as a natural reservoir with access to blood circulation and the capacity to enable solid forms to reach all parts around an infected area make intrapocket delivery systems desirable [15]. Commercially approved devices available on the market include Atridox^®^, Actisite^®^, Arestin^®^, Periostat^®^, and PerioChip^®^ [16]. Polymers used in the manufacturing of intrapocket drug delivery systems are either of natural origin, such as chitosan, cellulose, and alginate, or they are synthetic, such as poly(ε-caprolactone) (PCL), poly(D, L-lactide) (PLA), poly-(D,L-lactide-co-glycolide) (PLGA), poly-(vinylpyrrolidone) (PVP), and poly(vinyl alcohol) (PVA) [17,18,19]. Polymeric sustained-release drug delivery systems are designed and used for various aims, such as ocular [20] and periodontal [21] delivery.

PLGA is a favorable synthetic polyester that has high biocompatibility and good mechanical properties. Its disadvantages include hydrophobicity and limited bioactivity. These properties make it a potential polymer for use in commercial formulations to achieve better treatment goals [22,23,24]. PCL is also a synthetic polyester with good biocompatibility and nontoxicity in the body. In addition, it can produce appropriate fibers during electrospinning [25].

Nanofibers are modified-release formulations suitable for use as an intrapocket drug delivery system. Electrospinning is the most commonly used and efficient method for the preparation of nanofibers, and it has been applied at industrial and laboratory scales. It generally consists of a syringe pumping a polymer solution, a high voltage electric reservoir, and a conductive rotating collector [20,26,27,28]. Electrospinning has been used in the preparation of electrospun PLGA fibers for the local release of metronidazole in the treatment of periodontitis [29]. Amoxicillin-loaded electrospun nanofibers, produced by Ho et al., were reported to reduce inflammation and accelerate periodontal repair [30].

In this study, PLGA and PCL nanofibers containing amoxicillin and metronidazole were designed and evaluated for the treatment of periodontitis as intrapocket drug delivery systems. These formulations were then characterized for their physicochemical properties, safety and toxicity, and in vitro/in vivo evaluation. It was expected that these systems would achieve controlled release of both drugs.

## 2. Materials and Methods

### 2.1. Materials

Poly-(D-L-lactide-co-glycolide) (PLGA), poly(ε-caprolactone) (PCL), amoxicillin, metronidazole, dichloromethane (DCM), ethyl acetate (EA), ether, ketamine, xylazine, and ethanol were obtained from Merk Company (Darmstadt, Germany). Dulbecco’s Modified Eagle’s Medium (DMEM), fetal bovine serum, trypsin, sabouraud dextrose agar (SDA), sabouraud dextrose broth (SDB), soybean casein digest broth (SCDB), tryptic soy agar (TSA), thioglycolate culture medium, 3-(4,5-dimethylthiazol-2-yl)-2,5-diphenyltetrazolium bromide (MTT), and phosphate-buffered saline (PBS) were obtained from Bioidea Company (Tehran, Iran). Rats and rabbits were obtained from an internal animal laboratory in Kermanshah University of Medical Sciences. Human gingival fibroblasts (HGFs) were obtained from the Pasteur Institute (Tehran, Iran). The pH 7.4 used in the in vitro studies was prepared by using dihydrogen phosphate dodecahydrate and orthophosphoric acid, both purchased from Merck (Darmstadt, Germany).

### 2.2. Nanofiber Preparation

Two electrospinning solutions were prepared separately; one by dissolving 13% *w/v* of PLGA in a mixture of DCM/EA (9:1 *v/v* ratio) and the other by dissolving PCL at 10% *w/v* concentration in a similar solvent mixture. The solutions were stirred magnetically (300 rpm) at room temperature (25 °C) for 5 h to obtain clear and uniform solutions. Each polymeric solution was separated into two portions; metronidazole and amoxicillin were individually dissolved in each portion at 15% *w/w* of drug to polymers and stirred at 300 rpm for 2 more hours to obtain a clear solution. Finally, for each formulation, two electrospinning solutions were developed, one loaded with metronidazole and the other with amoxicillin. Blank nanofibers were prepared by dissolving polymers in the solvent system without a drug.

Through two individual electrospinning processes, PLGA and PCL nanofibers were prepared using a double-nozzle electrospinner. Each nozzle was filled with 10 mL of PCL/metronidazole and PCL/amoxicillin for the electrospinning of the PCL nanofibers. The same procedure was used in the case of the PLGA formulations (10 mL of PLGA/metronidazole and PLGA/amoxicillin). The temperature was kept between 25 to 30 °C for the PCL formulation and between 16 to 20 °C for the PLGA formulation using a temperature regulator. Other factors were kept the same for both samples. In the electrospinning process, a maximum voltage of 17 kV and a drum rotation rate of 250 rpm were applied. The needle was swept between distances of 110 to 190 mm from the baseline. A distance of 100 mm between the needle and the drum was also applied. The injection took place at a rate of 1 mL/h. Nanofibers were collected on the drum and stored in a refrigerator (2–8 °C) till required.

### 2.3. Physicochemical Studies

#### 2.3.1. Surface Morphology Determination by Scanning Electron Microscopy (SEM)

SEM (KYKY, EM-6200, Beijing, China) was used as a method to observe the surface morphology, uniformity, and structure of the nanofibers. Small pieces of the nanofibers were separated and sputter-coated with a thin layer of gold. The surface characteristics were examined under a vacuum and at 30 kV accelerating voltage. Various magnifications were used to ascertain the different morphologies.

#### 2.3.2. Chemical Structure Investigation by Fourier-Transform Infrared Spectroscopy (FTIR)

FTIR spectroscopy (Shimadzu IR PRESTIGE-21, Kyoto, Japan) was used to determine any interactions or changes at the molecular level of the starting materials used in the preparation of the nanofibers and also for the nanofibers themselves. A comparison of the nanofibers’ spectra can provide information about possible interactions. A small amount of the appropriate sample (drug, polymer, or nanofiber) was mixed with KBr and then compressed by an FTIR manual press under a pressure of 10 tons for 2–3 min. The samples were scanned between 400–4000 cm^−1^.

#### 2.3.3. Tensile Strength Measurement

The tensile strength properties of the nanofibers were determined using a tensiometer (Santam-STM5, Tehran, Iran). This method indicates the resistance of the nanofibers against an external force when placed between two clamps. The prepared nanofibers were cut into 2 × 2 cm^2^ pieces and placed in the tensiometer apparatus for strength measurements. The samples were pulled at a 1 mm/min stretching rate.

#### 2.3.4. Sterility Test

The sterility of nanofibers was studied by placing 20 mg of blank nanofibers into SDB, SCDB, and thioglycolate culture medium designed for fungal and aerobic and anaerobic bacterial growth. After 7, 14, and 21 days of incubation at 25 °C for the fungi and 37 °C for bacteria, the culture media were evaluated for possible occlusion due to microorganism growth compared to positive (cultured microorganisms) and negative (pure culture media) control samples.

#### 2.3.5. Bacterial Culture

*Escherichia coli* and *Staphylococcus aureus* were selected for this study as anaerobic and aerobic microorganisms, respectively. They were activated in TSA culture media for 24 h and incubated at 37 °C. The antibacterial activity of the nanofibers was assessed using the agar diffusion test. Disks of 5 mg of the formulation were measured and sterilized for 30 min under UV light at room temperature (25 °C) and placed on the cultured media. This was then incubated for another 24 h at 37 °C and the diameter of the inhibition zone around the nanofiber disks was measured.

#### 2.3.6. In Vitro Release Study

In vitro drug release was evaluated using the Franz diffusion cell method. A specific amount of nanofibers (20 mg, equivalent to 3 mg amoxicillin and 3 mg metronidazole) were cut and put into a pocket made of the diffusible cellulose dialysis membrane (cut-off: 12,000 Daltons). The membrane was then kept in a release medium containing 25 mL of phosphate buffer (pH = 7.4) at 37 °C stored in a shaker incubator with a rotation rate of 100 rpm. At certain predetermined time intervals, 5 mL samples were collected from the release medium for drug content analysis using HPLC (see Section 2.3.8). The samples withdrawn from the diffusion cells were replaced by the fresh medium (5 mL) and kept at 37 °C to keep the total volume constant in the Franz cells and also to maintain sink conditions throughout the test.

#### 2.3.7. Kinetics of Drug Release

The drug release data were fit to different kinetics models, including zero-order, first-order, Higuchi, and Korsmeyer–Peppas models. The model with the highest correlation coefficient was chosen as the best-fitted kinetic model. The equations used were as follows:

Zero-order release:

Qt = K_0_ t (1)

First-order release:

Ln Qr = −K_1_t + Q_0_ (2)

Higuchi model:

Qt = K_H_t^0.5^ (3)

Korsmeyer–Peppas:

Qt = K_p_t^n^ (4)

In these equations, Qt is the amount or percentage of drug released at time t and Qr and Q_0_ denote the remaining drug in the formulation (unreleased) and the initial amount of drug in the formulation respectively. K_0_, K_1_, K_H_, and K_p_ are constant drug releases and n in Equation (4) is the release exponent and indicates the mechanism of drug release.

#### 2.3.8. HPLC Analysis

Standard solutions with defined concentrations were prepared by dissolving drugs (amoxicillin and metronidazole) in distilled water at a stirring speed of 300 rpm at room temperature for 2 h to complete dissolution. Standard solutions (20 µL) were injected into the HPLC apparatus, where the mobile phase contained acetonitrile:phosphate buffer (1:1 *v/v* ratio; the pH of the mixture was adjusted to 4) at a 1 mL/min injection rate with the detection wavelength set at 220 nm. The HPLC analytical column (5 µm) Perfectsil ODS-2 (250 × 4 mm^2^; MZ Analysentechnik) was utilized. A twenty microliter sample loop was applied with retention times of 1.733 and 2.317 min for amoxicillin and metronidazole, respectively. Data were analyzed using the Excel program (Microsoft Office 2016). Regression equations were constructed based on the standard solutions to calculate the concentrations of drugs in the samples withdrawn from the Franz diffusion cell.

#### 2.3.9. Cytotoxicity Evaluation

Three pieces of nanofibers cut to ensure varying concentrations of 1000, 500, and 250 µg/mL of each drug (metronidazole or amoxicillin) were incubated in 1 mL of DMEM in sterile conditions for 48 h. The supernatant was then separated and used as an extract for direct cell contact.

HGFs were cultured in sterile plates in DMEM medium and incubated for 24 h at 37 °C in an incubator. The supernatant from the DMEM, which was initially incubated with the formulated nanofibers, was added to cultured cells and incubated for another 24 h in standard conditions for further evaluations.

The cultured cells were seeded into 96-well plates. Cell viability of human gingival fibroblasts was evaluated with the MTT test. The culture medium was discarded and plates were washed with PBS. Trypsin was added to free cell attachments and 10 µL of MTT solution (5 mg/mL) was then added to the wells. Control samples were prepared by adding DMSO to cultured cells for full cell inhibition. Plates were incubated for 4 h in an incubator at 37 °C and the absorbance of these samples was measured by UV spectrophotometry at a wavelength of 570 nm. The cell viability percentage was calculated by the following equation, in which C is the absorbance of the test samples and A is the absorbance of the control samples:

Cell viability (%) = C/A × 100(5)

#### 2.3.10. In Vivo Drug Release Test

Due to the small sizes of the oral cavity in animal models such as rat and rabbit models, which tend to be the most popular models for in vivo studies of intrapocket formulations, a replacement method was adopted [31]. This method included subcutaneous implantation of the drug preparation in the dorsal part of a rat model to evaluate the biocompatibility of the formulation with the tissue and its therapeutic performance. The proposed method has been previously utilized in independent studies. Xue et al. [32] used this method for the evaluation of antimicrobial intrapocket implants. Yadav et al. [33] designed guided tissue regeneration/guided bone regeneration (GTR/GBR) anti-infective formulations for periodontitis management and also adopted the same methodology for the evaluation of their formulations.

To sterilize the nanofibers for in vivo testing, the formulated nanofibers were cut into 25 mg pieces (equivalent to 3.75 mg of each drug) and were subjected to UV light for 30 min before use. Twelve adult male rats (200–250 g weight) were chosen and treated in standard feeding and nursing conditions. They were anaesthetized using an injection of 10% *v*/*w* ketamine. They were shaved on the dorsal part and two subcutaneous pockets of 1 × 1 cm^2^ were cut. Each pocket was used for the implantation of one formulation. The nanofibers cut into tiny pieces were implanted and pockets were closed by 000 size sutures. After 24, 48, 72, 96, 120, 144, 168, 216, 264, and 312 h, nanofiber samples were taken out from the subcutaneous pockets and prepared for HPLC method analysis.

The implanted nanofibers were explanted from the rats’ bodies after the rats were euthanatized by ether. The nanofiber samples were then washed with distilled water and dried in an oven at 60 °C for 30 min to obtain the primary weight of the nanofibers. The nanofibers were then dissolved in the least amount (~1 mL) of solvent mixture (DCM/EA (9:1 *v/v* ratio)) and characterized using the HPLC method, as previously described in Section 2.3.8.

#### 2.3.11. In Vivo Biocompatibility

In this experiment, the nanofibers were subcutaneously implanted in two animal models (rats and rabbits) with the procedure described in Section 2.3.10. After the implantation of the nanofibers at 1 and 3 week intervals, tissue samples were collected and fixed in 10% *w/v* formalin for 24 h and gradually dehydrated using ethanol. The standard procedure of dehydration was adopted, including the placement in graded series of ethanol (in PBS) for 15 min each (30%, 50%, 70%, 90%, and 100%) (n = 3). Sections were paraffined and cut into 6 µm slides. These were then colored using hematoxylin and eosin (H&E). Slides were observed with a light microscope and evaluated for the existence of inflammatory cell lines [34].

## 3. Results and Discussion

### 3.1. Surface Morphology

**Figure 1:**
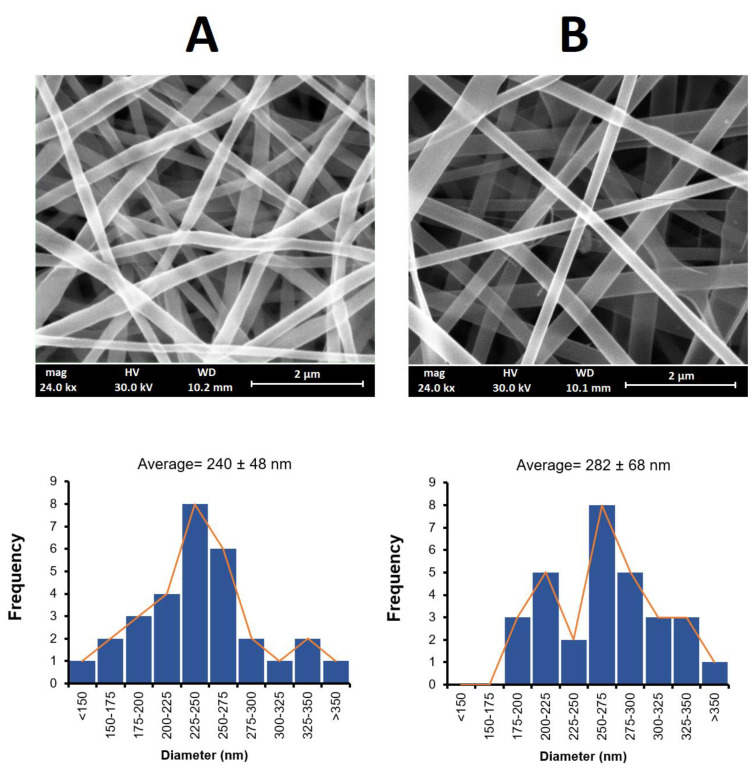
SEM images of PLGA (**A**) and PCL (**B**) nanofibers and histograms of their respective size distributions.

The nanofibers obtained using the electrospinning method had a white to yellowish color with smooth surface structures, as shown in Figure 1. The SEM images of these nanofibers were found to have a uniform structure with no beading. A similar morphology for PLGA and PCL nanofibers has been reported elsewhere [35,36]. Figure 1 also shows the size distribution of the obtained nanofibers. The mean fiber diameter was measured as 240 ± 48 nm for the PLGA nanofibers and 282 ± 68 nm for the PCL nanofibers. The larger diameter obtained for the PCL fibers may have been due to the higher viscosity of the electrospinning solution arising from the higher molecular weight of PCL (Mw = 80,000) compared to PLGA (Mw = 20,000), despite the different %*v/v* used [37].

### 3.2. FTIR Spectroscopy

**Figure 2:**
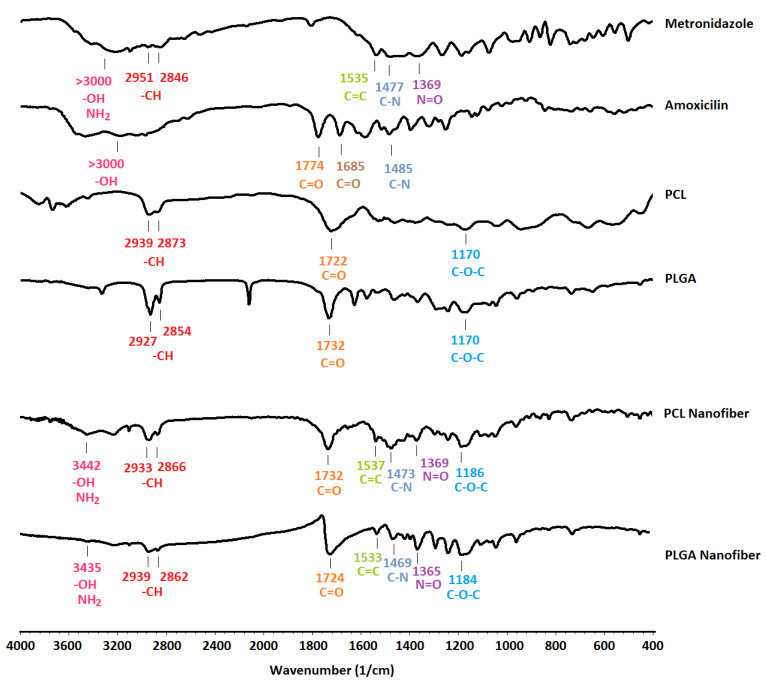
FTIR spectra and characteristic peaks of metronidazole, amoxicillin, PCL, PLGA, and the formulated nanofibers.

A comparison of the nanofiber spectra for both formulations with the individual excipients used in the preparation of the nanofibers showed no interactions (Figure 2). The characteristic peaks of the polymers and drugs were present in the spectra of the nanofibers (Figure 2). The spectrum of the PCL nanofibers indicated multiple characteristic peaks corresponding to the functional groups of drugs and polymers. The peak at 3442 cm^−1^ was assigned to the hydroxyl and amino groups of amoxicillin and metronidazole. The absorption bands at 2933 cm^−1^ and 2866 cm^−1^ were related to the symmetric and asymmetric C-H stretching vibrations of metronidazole and PCL, respectively. The 1732 cm^−1^ peak was attributed to the stretching vibrations of the carbonyl (C=O) groups of PCL and amoxicillin, whilst the peak at 1721 cm^−1^ corresponded to the carbonyl group of the beta-lactam ring. The peak at 1685 cm^−1^ merged with a wide 1721 cm^−1^ peak was indicative of the C=O group adjacent to the NH_2_. The C=C stretching vibration of metronidazole appeared at 1537 cm^−1^. The C-N stretching of metronidazole and amoxicillin was also present at 1473 cm^−1^. The stretching of the N=O group in the metronidazole structure was detectable as a peak at 1369 cm^−1^. The peak at 1186 cm^−1^ was related to the C-O-C group of PCL.

In the case of the PLGA nanofibers, a wide peak at 3435 cm^−1^ was assigned to the -OH and NH_2_ groups. Symmetrical and asymmetrical stretching vibrations of C-H appeared as two characteristic peaks at 2939 cm^−1^ and 2862 cm^−1^. The carbonyl stretching vibrations of the PLGA and the amoxicillin beta-lactam ring appeared at 1721 cm^−1^. The peak at 1685 cm^−1^ was a result of the C=O groups adjacent to the NH_2_ and was merged with the side peak. The peak at 1469 cm^−1^ was assigned to the C-N stretching of both drugs. The characteristic N=O stretching peak was detectable at 1365 cm^−1^ in the peak of the PLGA nanofibers. The C-O-C stretching appeared at 1184 cm^−1^.

In conclusion, there were no significant changes, such as decreases in the intensity or elimination of characteristic peaks. It can therefore be concluded that no significant interactions occurred between the drug and the polymers.

### 3.3. Tensile Strength

The tensile strength results showed that the PLGA nanofibers were more resistant to tension than the PCL nanofibers when external stress was applied. This is due to the high inherent stiffness of PLGA [38]. Chou et al. reported PCL and PLGA fibers to have tensile strengths between 2-4 MPa, which is in agreement with the results of this current study. It has also been reported that drug-loading can lead to decreased tensile stress compared to blank fibers. The PLGA nanofibers showed a tensile strength of 2.9 MPa with an elongation at break of around 8.5%, whilst in the case of the PCL nanofibers these values were 1.9 MPa and 4.5%, respectively. The tensile strength of nanofibers is highly related to the porosity and to the width-to-length ratio of the mat and each strain of fiber. An increase in the porosity or a reduction of the width-to-length ratio therefore leads to a reduction in the tensile strength of the nanofibers, even if the polymeric fibers have an appropriate intrinsic stiffness [39]. The prepared PLGA nanofibers demonstrated suitable tensile strength through the optimization of the electrospinning conditions and solvent system and thus resulted in an appropriate level of porosity and width-to-length ratio.

### 3.4. Sterility Test

The sterility of the blank nanofibers (without drug) was evaluated by placing the sterilized nanofibers into the selected culture media to observe possible microorganism growth. After incubation time (7, 14, and 28 days), the test samples were just as clear as the negative controls in comparison to turbid positive controls. No microorganism growth was detected in all the culture media, indicating the sterility of the prepared nanofibers. The sterility was thus achieved by performing the whole preparation process under aseptic conditions and storing the samples in a sterile environment.

### 3.5. Microbial Assay

**Figure 3:**
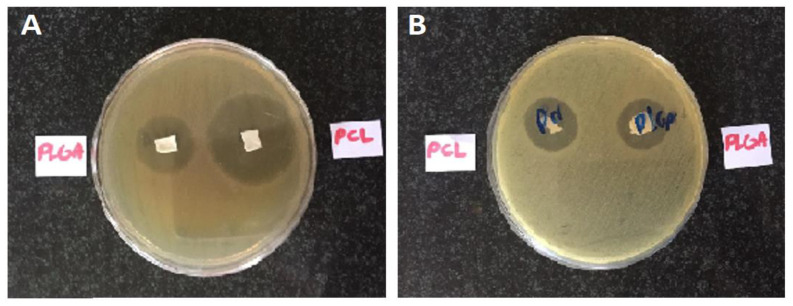
The inhibition growth zones and antimicrobial efficacy of the prepared nanofibers against *S. aureus* (**A**) and *E. coli* (**B**).

Incubated bacterial cultures containing sterilized nanofibers were detected for the growth inhibition zone, which represents an inhibition effect of the formulated nanofibers on the studied microorganisms. The diameter of the growth inhibition zones was measured to be 11 ± 1 mm for the PCL nanofibers and 12 ± 1 mm for the PLGA nanofibers on *E. coli* and 23 ± 2 mm for the PCL nanofibers and 12 ± 1 mm for the PLGA nanofibers on *S. aureus*. It can thus be concluded that these nanofibers have antimicrobial efficacy against both Gram-positive and Gram-negative bacteria. Figure 3 depicts the inhibition growth zones of the nanofibers against bacteria.

Haider et al. prepared PLGA/CuO hybrid nanofiber scaffolds to determine their inhibitory effect on microorganisms involved in oral pathogenesis [40]. The authors showed the effectiveness of the PLGA nanofibers on Gram-positive bacteria (*Staphylococcus aureus*) and Gram-negative bacteria (*Escherichia coli*).

### 3.6. In Vitro Drug Release

**Figure 4:**
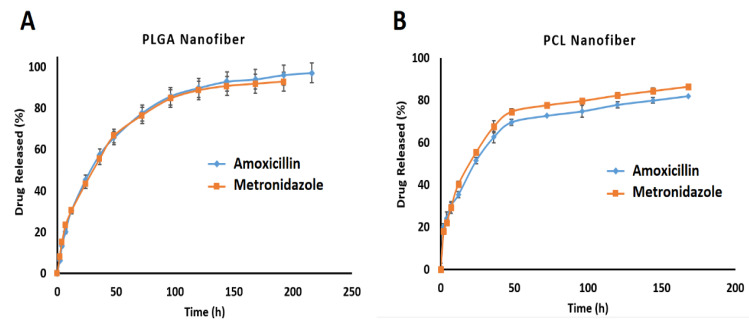
The percentage of cumulative drugs released from (**A**) PLGA nanofibers and (**B**) PCL nanofibers in phosphate buffer (pH = 7.4) at 37 °C in vitro measured by HPLC.

The amount of drug release was quantified by HPLC. Using the drug release data, the release profiles of the drugs from the nanofiber formulations were plotted and are shown in Figure 4A,B.

All formulations released most of the drugs within 180 h. These release profiles showed two phases of drug release. Almost 50% of the drug was released within the first day followed by a gradual release over 6–8 days. For example, the PLGA formulation released 45.28 ± 1.39% of the amoxicillin and 43.30 ± 4.16% of the metronidazole within the first 24 h whereas the PCL nanofibers released 55.34 ± 1.37% of the metronidazole and 51.32 ± 1.41% of the amoxicillin over the same period. It seems that, in the second phase, the release of drugs from the PLGA nanofibers was faster than the drug release from the PCL nanofibers. For instance, almost 80% of both the amoxicillin and metronidazole were released within 78 h, whereas in the case of the PCL nanofibers the time taken to release 80% of the amoxicillin and metronidazole was 145 h and 97 h, respectively.

The more gradual and controlled release of drugs from the PCL nanofibers (196 h for the release of the metronidazole and 216 h for the release of the amoxicillin) compared to the PLGA nanofibers (168 h for the release of both drugs) may have been due to the higher hydrophobicity of PCL compared to PLGA [41]. This high hydrophobicity may have led to a decreased degree of swelling and consequently a slower release profile.

Tseng et.al prepared PLGA nanofibers containing lidocaine for topical delivery which showed a controlled release pattern for 14 days in the laboratory setting [42]. Dias et.al also prepared PCL nanofibers containing oxytetracycline and zinc oxide designed for the treatment of periodontitis. The in vitro drug release time was determined to be at least 120 h [43].

### 3.7. Drug Release Kinetics

Table 1 shows the parameters of the different kinetic models to which the release data were fitted. The Higuchi model was the best-fitted model for the drug releases from the PLGA and PCL nanofiber formulations. These results suggest that the release mechanism was dominantly ruled by the diffusion phenomenon. In other words, due to the high hydrophobicity of both PCL and PLGA, the release profiles were more related to diffusion than degradation and resulted in a Fickian diffusion mechanism and a two-step release process [44].

The Higuchi release constants were calculated for both formulations and the results indicated that their values were very close to each other. The K_H_ for metronidazole release from the PLGA and PCL formulations was determined to be 0.1019 and 0.1069, respectively, whilst the K_H_ for amoxicillin release from the PLGA and PCL formulations was 0.1079 and 0.0916, respectively.

### 3.8. Cytotoxicity Evaluation

**Figure 5:**
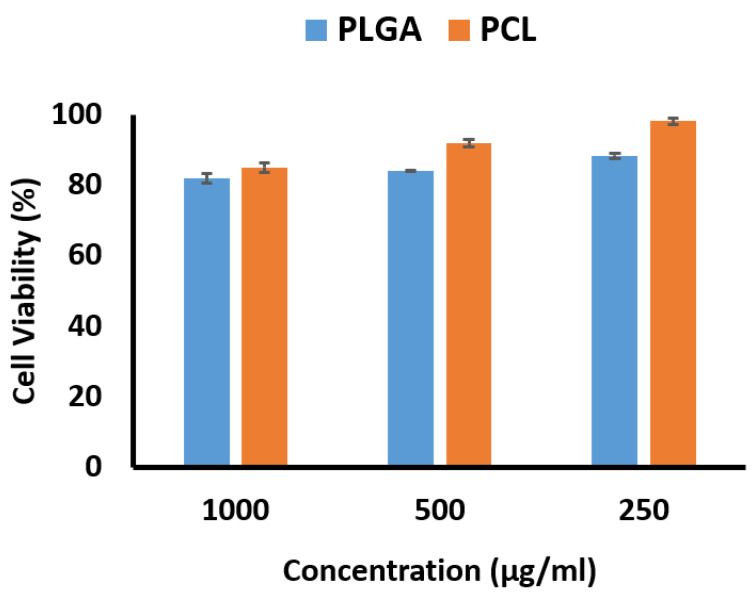
Cell viability percentages of PCL and PLGA nanofibers at different concentrations (250–1000 µg/mL).

The percentages of cell viability based on concentration as determined by the MTT method are shown in Figure 5. The cell viability percentage for the PCL nanofibers was higher than for the PLGA nanofibers, with both exhibiting more than 80% cell viability as noncytotoxic preparations. This is an interesting result, as the effects of PLGA nanofibers on mesenchymal cells have been investigated by Sfakis et al., and they observed the cell viability as measured by the MTT method to be 40% [45]. Lim et al. reported cell survival at 80% when PCL nanofibers were investigated in a human skin fibroblast cell line [46].

### 3.9. In Vivo Drug Release

**Figure 6:**
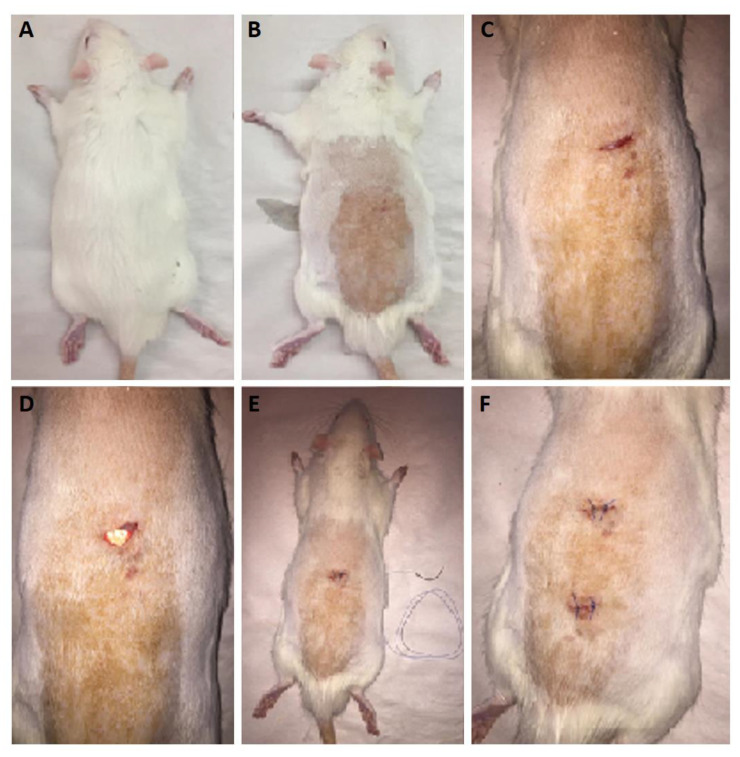
Surgical implantation of nanofibers into rat skin. (**A**) Healthy rat before the procedure; (**B**) anesthetization and shaving of the dorsal part; (**C**) cutting the subcutaneous pocket for implantation; (**D**) placement of nanofibers in the pocket; (**E**) closure of the pocket; (**F**) suture.

**Figure 7:**
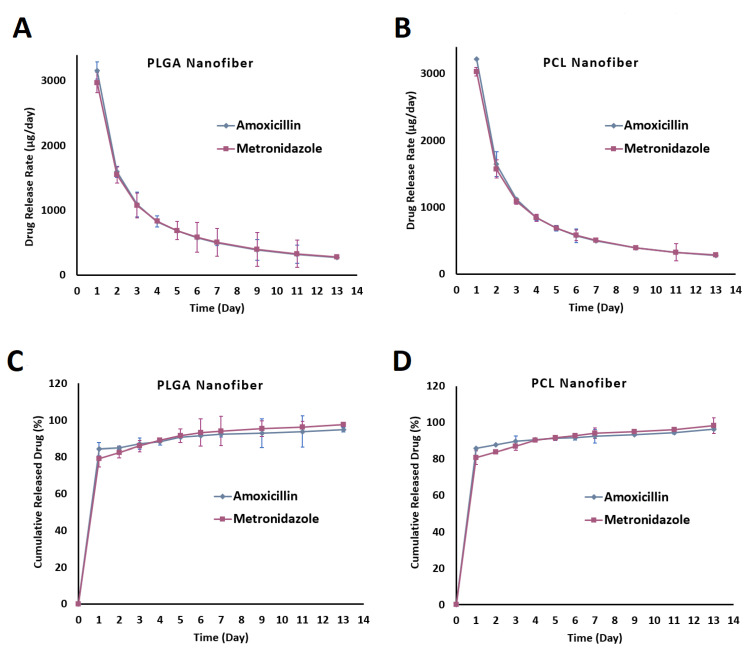
Drug release rate (µg/day) from PLGA (**A**) and PCL (**B**) nanofibers and cumulative percentages of released drugs from PLGA (**C**) and PCL (**D**) nanofibers in the subcutaneous tissue of rats during the in vivo study.

It is important to carry out in vivo release studies, as these can provide more realistic data on the drug release from the nanofibers. As previously mentioned in the methodology section, the nanofibers were implanted surgically in subcutaneous pockets after sterilization. The surgical procedure in the rat model is depicted in Figure 6. The in vivo release profiles for both drugs are shown in Figure 7.

Figure 7A,B show the rate of drug release during the 13 days of implantation. Figure 7 shows that both sets of nanofibers presented the maximum release rate in the first day of implantation. The PLGA nanofibers released amoxicillin at 3160.66 ± 131.87 µg/day and metronidazole at 2972.32 ± 157.31 µg/day in the first 24 h. The PCL nanofibers released amoxicillin and metronidazole at 3215.90 ± 89.08 µg/day and 3032.21 ± 130.57 µg/day, respectively, in the same period. The release rate decreased after the first day of implantation to a range between 200 and 1600 µg/day. Both formulations demonstrated almost similar in vivo release rate profiles.

The cumulative percentages of drugs released are shown in Figure 7C,D. Both PLGA and PCL nanofibers released almost 90% of both drugs within 4–5 days. The prolonged-release profiles of the drugs with the nanofibers make these systems suitable for the controlled delivery of these antibiotics for dental infection. These systems can act as a reservoir for intrapocket drug delivery systems, providing effective local concentrations of drugs along, and reduce the frequency of drug administration from three times a day in a systemic manner to once weekly as local delivery, hence increasing patient compliance. In a similar study, nanocomposites containing metronidazole and curcumin designed for dental administration showed positive effects on dental tissue regeneration [47].

### 3.10. In Vivo Biocompatibility

**Figure 8:**
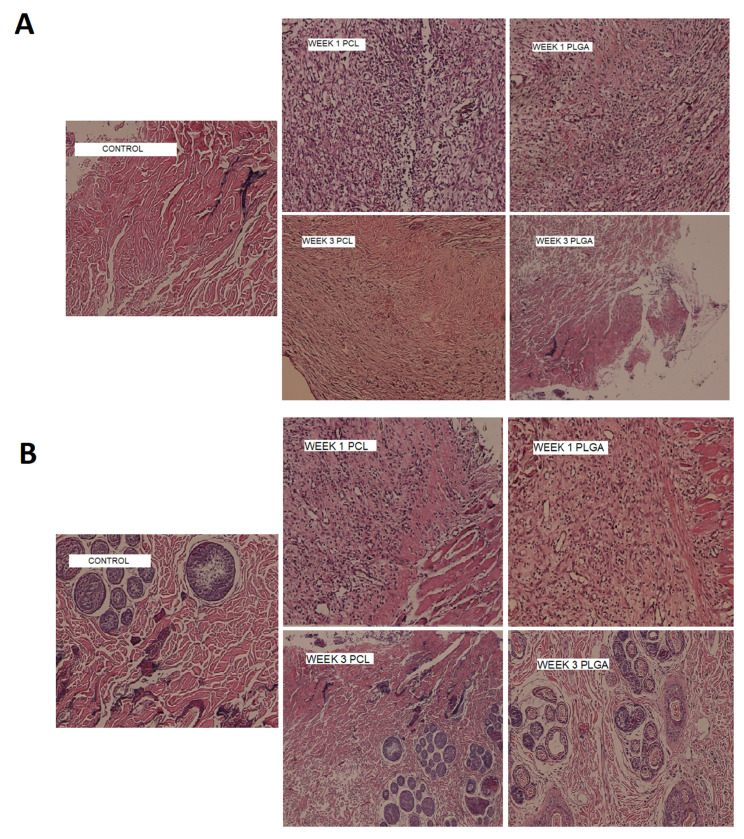
Histopathological changes of subcutaneous tissues after implantation of nanofibers and comparison with control group over 3 weeks in a rat model (**A**) and rabbit model (**B**).

Nanofibers were surgically implanted into subcutaneous pockets in rat and rabbit models for biocompatibility assessments. The comparison of the first- and third-week samples with the control slides in the rat and rabbit models for the presence of neutrophils, macrophages, and giant cells as inflammatory markers was undertaken (Figure 8). Figure 8 shows that, after three weeks, the degree of inflammation was reduced compared to the first week of implantation in both models. The PLGA nanofibers were more biocompatible compared to the PCL nanofibers, and the rabbits showed fewer inflammatory reactions to implanted nanofibers compared to the rats. In a previous study in which metronidazole was incorporated into PCL nanofibers and the toxicity of the nanofibers in a rabbit animal model was studied, the results indicated high biocompatibility with tissue after 1 and 7 days of implantation [32].

## 4. Conclusions

Nanofibers are one of the most promising formulations for use in modified release systems for direct drug delivery that are made of polymers that are compatible with body tissues. In this study, PLGA and PCL nanofibers containing metronidazole and amoxicillin were successfully manufactured and characterized. These nanofibers were able to release their drug contents in a controlled manner and keep the concentration of the drug at the right level in the local area to treat the infection. SEM studies of the nanofibers showed that they had smooth surfaces and could be used in an intrapocket drug delivery system. Solid-state analysis ruled out any interactions between the drugs and the polymers used in the nanofibers’ manufacture. Nanofibers were also studied for cytotoxicity and biocompatibility in both cell culture and animal model experiments. The promising results suggest the possibility of using these new formulations as an effective therapeutic method for the treatment of periodontitis. In vitro results showed good controlled drug release for the treatment goals. Drugs were released at a suitable concentration over a period of 7 to 10 days in an animal model. The prolonged-release profiles indicate that these systems could increase patient compliance by reducing the administration frequency. It can therefore be concluded that these systems have the potential to enter clinical studies and to be used as routine effective treatment modalities against periodontitis.

## Figures and Tables

**Table 1:** The correlation coefficients obtained by fitting the drug release data from the nanofibers to the zero-order, first-order, Higuchi, and Korsmeyer–Peppas models.

Formulation	Correlation Coefficient			
	Zero-Order	First-Order	Higuchi	Korsmeyer–Peppas
PLGA-Amoxicillin	0.9584	0.9934	0.9986	0.9816
PLGA-Metronidazole	0.9707	0.9944	0.9976	0.9870
PCL-Amoxicillin	0.9752	0.9946	0.9964	0.9880
PCL-Metronidazole	0.9598	0.9961	0.9966	0.9940

## Data Availability

Not applicable.
